# Private Receptions

**Published:** 1867-06

**Authors:** 


PRIVATE RECEPTIONS.

On Wednesday, May 8th, at 4 P.M., by Mr. and Mrs. George H. Pendleton, at their residence in Clifton. Carriages will leave Hopkins' Hall at $2\frac{1}{2}$ P.M.

On Thursday evening, May 9th, at 8 P.M., by Mr. and Mrs. Larz Anderson, north-east corner of Pike and Third Streets.

On Thursday evening, May 9th, by Hon. C. F. Wilstach, at 8 P.M., No. 559 West Court street.

On Thursday evening, May 9th, at 8 P.M. by Dr. and Mrs. George Mendenhall, No. 197 West Fourth street.

On Thursday, at 12 M., May 9th, steamboat excursion in honor of the American Medical Association, by the City Council of Cincinnati. The steamer America will leave the landing at Vine street, at 12 M. precisely.

The Chief of the Fire Department, Enoch Megrue, Esq., will give an exhibition of the Steam Fire Engines, Thursday morning, at 9 A.M.

Messrs. W. P. & F. P. Anderson, proprietors of Longworth's Wine House, will receive the delegates at 9 A.M., Friday, May 10th, at No 113 East Sixth street.

Excursion to Longview Lunatic Asylum. Reception by the Superintendent, Dr. O. M. Langdon, Friday, May 10th, at 4 P.M.

A special train will leave the Cincinnati, Hamilton & Dayton depot at $3\frac{1}{2}$ P.M., for the Asylum.

